# A tRNA-mimic Strategy to Explore the Role of G34 of tRNA^Gly^ in Translation and Codon Frameshifting

**DOI:** 10.3390/ijms20163911

**Published:** 2019-08-11

**Authors:** Aurélie Janvier, Laurence Despons, Laure Schaeffer, Antonin Tidu, Franck Martin, Gilbert Eriani

**Affiliations:** Architecture et Réactivité de l’ARN, Université de Strasbourg, Centre National de la Recherche Scientifique, Institut de Biologie Moléculaire et Cellulaire, 2 allée Konrad Roentgen, 67084 Strasbourg, France

**Keywords:** genetic code, tRNA, glycine codon, translation, IRES element, frameshifting

## Abstract

Decoding of the 61 sense codons of the genetic code requires a variable number of tRNAs that establish codon-anticodon interactions. Thanks to the wobble base pairing at the third codon position, less than 61 different tRNA isoacceptors are needed to decode the whole set of codons. On the tRNA, a subtle distribution of nucleoside modifications shapes the anticodon loop structure and participates to accurate decoding and reading frame maintenance. Interestingly, although the 61 anticodons should exist in tRNAs, a strict absence of some tRNAs decoders is found in several codon families. For instance, in Eukaryotes, G34-containing tRNAs translating 3-, 4- and 6-codon boxes are absent. This includes tRNA specific for Ala, Arg, Ile, Leu, Pro, Ser, Thr, and Val. tRNA^Gly^ is the only exception for which in the three kingdoms, a G34-containing tRNA exists to decode C3 and U3-ending codons. To understand why G34-tRNA^Gly^ exists, we analysed at the genome wide level the codon distribution in codon +1 relative to the four GGN Gly codons. When considering codon GGU, a bias was found towards an unusual high usage of codons starting with a G whatever the amino acid at +1 codon. It is expected that GGU codons are decoded by G34-containing tRNA^Gly^, decoding also GGC codons. Translation studies revealed that the presence of a G at the first position of the downstream codon reduces the +1 frameshift by stabilizing the G34•U3 wobble interaction. This result partially explains why G34-containing tRNA^Gly^ exists in Eukaryotes whereas all the other G34-containing tRNAs for multiple codon boxes are absent.

## 1. Introduction

The process of translation is the last stage in the genetic information transfer and it depends upon the correct matching between mRNA codons and corresponding tRNA anticodons within the ribosomal complex. High fidelity of tRNA selection is crucial in the expression of genetic information. On the ribosome, each codon of the mRNA has to be accurately and efficiently recognized by cognate tRNAs among a large repertoire of non-cognate tRNAs. Aminoacylated tRNAs (aa-tRNAs) are delivered to the ribosomal A site as a ternary complex with elongation factor EF-Tu and GTP. Incorrect tRNA can be rejected either before or after GTP hydrolysis. During the initial selection step, the ternary complex of aa-tRNA with EF-Tu–GTP (in procaryotes) binds to the ribosome and forms a readily reversible complex that dissociates rapidly when there is no codon/anticodon match. This step occurs without GTP hydrolysis. Most non-cognate tRNAs are rejected by this first sieve with essentially no cost with respect to GTP hydrolysis [1]. Discrimination of near-cognate ternary complexes is enhanced by a proofreading step that requires a more complex mechanism with the hydrolysis of GTP in EF-Tu. After GTP hydrolysis, EF-Tu undergoes a dramatic conformation change, thereby loosing its affinity for aa-tRNA. The aminoacyl end of aa-tRNA is thus free to move into the peptidyl transferase centre on the 50S subunit (accommodation), where it takes part in peptide bond formation. Alternatively, after GTP hydrolysis, near-cognate aa-tRNA with imperfect base pairing dissociate from the ribosome. Overall, discrimination of correct and incorrect substrates is achieved in two consecutive steps, initial selection and proofreading which are separated by GTP hydrolysis. All these events between the initial binding and accommodation of the cognate aa-tRNA have been dissected into several distinguishable steps (reviewed in [2,3]).

Although the process of tRNA selection on the ribosome seems to be universal, examples of decoding of codon-anticodon pairs that deviate from the canonical process have been described. Dicistroviruses such as cricket paralysis virus (CrPV) contain between their two ORFs, an IRES element called intergenic region (IGR) that mimics the structure of tRNA presenting to the ribosome a pseudo codon-anticodon triplet. The IGR forms a pseudo knot (PKI), which mimics the codon-anticodon nucleotides. It enters the A-site, translocates to the P-site, and initiates translation on the next codon, a non-AUG initiation codon [4,5]. Translation initiation is independent from the sequence of the triplet present in the PKI and can occur with a stop codon [5]. However, when the pseudo codon-anticodon triplet is disrupted by a mutation in the PKI sequence, the IGR is unable to initiate translation on the downstream ORF [5]. In short, the IGR mediates ribosome hijacking in achieving a sophisticated molecular mimicry of tRNA and mRNA. For that, the IGR adopts a modular structure composed of 6 stem-loops and 3 pseudo knots, balanced between rigidity and flexibility in order to optimally interact with the ribosome and elongation factor eEF2 during translocation [6,7,8,9]. It was shown that PKI is recognized by the ribosome in the same way as a cognate mRNA-tRNA codon-anticodon duplex. Stem loops IV and V of the IGR play a significant role during this process by interacting with the head of the ribosome to restrict its movement as observed during canonical translation [9,10,11]. Once PKI has entered the P-site, the contacts with stem loops IV and V are disrupted, leaving the A-site free to accept the first aminoacylated tRNA complexed with elongation factor eEF1A [12]. After a second translocation, the IGR mimics a canonical acceptor stem of an E-site tRNA [13]. Altogether, the data show that PKI of the IGR follows the conventional molecular mechanisms of tRNA discrimination by sensing the codon/anticodon duplex in the A site and the subsequent step of translocation in the P site [9]. Following 80S assembly, the eEF2-mediated translocation of the IGR in the P-site results in an unstable intermediate that is captured by binding of the elongator tRNA in complex with eEF1A. At this step, initiation can occur in the main frame (0 frame) and also after frameshifting in the +1 frame, according to the arrival rate of the first tRNA [14]. Usually, frameshifting during decoding generates much more drastic effects than missense errors that change a single amino acid residue (estimated to be in the range 6 × 10^−4^–5 × 10^−3^) [15]. It occurs when the ribosome recruits an aa-tRNA out of frame with respect to the frame in which it was initiated. The most common of these events require the ribosome to shift to a codon overlapping a codon in the normal frame. A shift of one nucleotide toward the 5′ end of the mRNA generates a (−1) frameshifting, whereas a shift toward the 3′ end generates a (+1) frameshifting. Shifted ribosomes that cannot finish the normal protein. They no longer see the correct codons, and normally rapidly encounter a termination codon. The importance on the protein function is usually consequent because of the sequence change and premature stop. Although the spontaneous frequency of frameshift errors is below missense errors, it can be considerably increased by additional factors such as slippery sequences or ribosomal pausing on hungry codons that modify the codon-anticodon realignment [16]. In addition, some frameshifts are programmed by primary and secondary sequence elements either preceding or following the shift site [16,17,18].

Multiple types of molecules in the translational machinery can promote translational frameshifting, including ribosomal RNA, ribosomal proteins, and translation elongation factors [16]. A common molecule affecting the frame maintenance is the tRNA. Frameshift suppressor tRNAs often harbour alterations of the loop of the anticodon but also normal 7-nt anticodon loops [19]. Changes in the tRNA base modifications such as N1-methylguanosine at position 37, are also able to induce frameshifting [20,21,22]. Recently, a structural study revealed that suppressor tRNASufA6 (tRNA^Pro^ with an expanded anticodon loop) is undergoing a rearrangement of the anticodon loop due to the extra nt at position 37.5 that destabilizes U32, thereby disrupting the conserved U32-A38 base pair [23]. Interestingly, the removal of the m_1_G37 modification of tRNA^Pro^ disrupted U32-A38 pairing in a structurally analogous manner, explaining the frameshifting effects previously observed [24]. Altogether, these data showed that the disruption of the U32–A38 base pair plays a significant role in the loss of grip in the correct frame. Phylogenetic analysis has shown that the 32–38 interaction is a fundamental feature of all tRNAs and is directly correlated to the strength of the codon–anticodon interaction (high vs. low GC base pairs) [20,21]. Modifying the 32–38 interaction dramatically affects tRNA discrimination of cognate vs. near-cognate codons both in vitro and in vivo [25,26].

Much of what we know about frameshifting induced by tRNAs has come from studies on suppressors of tRNA^Gly^_NCC_ and tRNA^Pro^_NGG_. Sequencing of suppressible frameshift mutations showed that they include the sequences CCC-N or GGG-N, resulting from expansion of proline (CCN) or glycine (GGN) codons. This allows the tRNA suppressor to frameshift from the original phase 0 to the shifted phase (+1) while keeping the codon-anticodon pairing. Glycine belongs to the GC-rich 4 codon boxes that establish strong base pairing with their corresponding anticodon. The structures of these tRNAs have been ‘tuned down’ during evolution to reduce and adjust their binding strength to the mRNA in the ribosome [27,28]. The adjustment structure is obtained by varying nucleotides forming the interaction 32–38 in the anticodon loop. These nucleotides should not form a Watson–Crick base pair or another interaction that would antagonize the canonical conformation of the codon/anticodon triplet in a correct helical orientation in the ribosomal decoding site. Without a selection for the preformed conformation of the anticodon loop, the strong codon*/*anticodon pairs would allow miscoding by binding to other G*/*C-rich near- or even non-cognate aatRNAs.

In eukaryotes, three tRNA^Gly^ isoacceptors are found with GCC, CCC and UCC anticodons. According to the wobble rules, tRNA^Gly^_UCC_ should be able to decode GGA and GGG codons. The tRNA^Gly^_GCC_ isoacceptor should decode GGC and GGU codons. Theoretically, these two tRNA species, that are the most abundant, should be able to decode the entire set of glycine codons, however, an additional tRNA^Gly^ with CCC anticodon is found to decode GGG codons [29]. Nucleotide U34 of tRNA^Gly^_UCC_ appears to be unmodified in higher eukaryotes, suggesting that it may also decode all 4 Gly codons by “superwobbling” [30]. Remarkably among eukaryotic tRNAs reading the 4 and 6 codon boxes, tRNA^Gly^ is the only one that exhibits a G34. Other tRNAs have a A34 that is modified in inosine to decode NNU, NNC and NNA codons (the later inefficiently [31]). Therefore, the decoding ability of G34 and I34 does not drastically differ and the reason of this dichotomy is not yet understood. Recently, an interesting correlation was described between the A34->I modification and the proofreading capacity of corresponding aminoacyl-tRNA synthetases (aaRS), the enzymes in charge of tRNA aminoacylation [32]. It was suggested that the proofreading activity would prevent continued sectoring of the genetic code utilizing the anticodon wobble position. The A34->I modification, found in tRNAs reading 4-codon boxes, would block its subdivision in two 2-codon boxes and thereby impact the evolution and sectoring of the genetic code [32]. Gly would escape to this trend, perhaps because glycyl-tRNA synthetase is highly specific for Gly and does not required proofreading activity. Therefore, Gly would not be concerned by codon reattribution and tRNA^Gly^ could use a G34 wobble and act as a ‘2-codon box reader’ without the risk of inducing the split of its 4-codon box [32]. In short, it seems that tRNAs^Gly^ bring together several features and properties that make them singular. They decode GC-rich codons, implying an adaptation of the anticodon loop structure to balance the energy of the codon-anticodon helix. They do not exhibit modification of A37, which is usually involved in reading frame maintenance. Many suppressors of tRNA^Gly^ that frameshift in +1 have been be selected [33] (and ref inside) and programmed ribosomal frameshifting on GGG codon in −1 frame is utilised by HIV-1 virus to modulate GagPol synthesis [34]. All these features suggest that tRNAs^Gly^ may play a general and significant role in the phenomenon of programmed ribosomal frameshifting, which regulatory role in both prokaryotes and eukaryotes is becoming increasingly clear [35,36,37,38] (and references therein).

In this context and to further explore the decoding and frameshifting properties of tRNA^Gly^ we performed the present study which combines experimental and bioinformatics approaches to carry out a systematic analysis of the Glycine codon usage in Eukaryotes and relationship with the 3′ adjacent nucleotide (nt+1). We found a clear distribution preference for a G over the 3 other nucleotides immediately after the GGU codon, from which we infer that this nucleotide likely causes an effect on translation. We assessed the effect of the four nucleotides at n+1 position in rabbit reticulocyte extracts by monitoring renilla luciferase activity of constructs programmed with the tetranucleotide GGU(N) in the two frames 0 and +1. To control both codon and anticodon pairings we used the internal ribosome entry site (IRES) from cricket paralysis virus (CrPV) that initiates using a pseudoknot structure mimicking a tRNA paired with the mRNA. We found that nt+1 regulates the frame selection by the IRES. Moreover, a G at +1 improves luciferase activity and favours the frame 0. Altogether, the results suggest that translation is modulated by the first nucleotide of the adjacent codon within the ribosome.

## 2. Results and Discussion

### 2.1. tRNAs^Gly^ Have Unusual Nucleotide 34 Distribution

**Figure 1:**
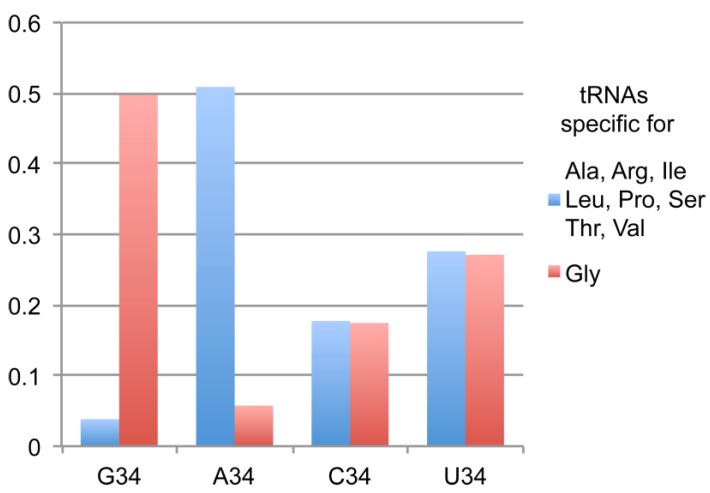
Distribution of nucleotide 34 in tRNAs specific for 3, 4 and 6 codon boxes according to the amino acceptor identity. The bars represent the usage of each nucleotide 34 in the subgroups containing tRNA^Gly^ alone or tRNAs specific for Ala, Arg, Ile, Leu, Pro, Ser, Thr and Val. G34 is overrepresented in tRNA^Gly^ and nearly absent in tRNAs specific for Ala, Arg, Ile, Leu, Pro, Ser, Thr and Val.

The current ‘genomic tRNA data base’ contains in the eukaryota domain 139,808 tRNA genes (http://gtrnadb.ucsc.edu/) [29]. Analysis of these data confirmed previous observations on the poor usage of G_34_NN anticodons by tRNAs specific of amino acids coded by 3, 4 and 6-codon boxes. Only 3063 sequences have been reported for the following amino acids: Ala, Arg, Ile, Leu, Pro, Ser, Thr and Val, which represents less than 4% of the total number of tRNA sequences (Table 1). Remarkably, tRNA^Gly^, which also belongs to the 4-codon box group, clearly escapes this global trend and 6636 sequences harbouring an anticodon G_34_CC are found in the database. In total, 13,347 sequences of tRNA^Gly^ have been reported in eukaryotes and the anticodon distribution is 767 for ACC, 2328 for CCC and 3616 for UCC in addition to the 6636 for GCC. Another surprising aspect of this distribution is the low number of genes for tRNA^Gly^ with ACC anticodons (only 767) compared to the other tRNA specific of the 3, 4 and 6 codon boxes. For instance there are 40,726 sequences exhibiting A34 in tRNAs specific for Ala, Arg, Ile, Leu, Pro, Ser, Thr and Val and only 767 sequences of tRNA^Gly^. It results a spectacular inversion of the usage of G34 versus A34 when comparing tRNA^Gly^ numbers with the other tRNAs specific for Ala, Arg, Ile, Leu, Pro, Ser, Thr and Val (Figure 1). Altogether, the data are suggesting that tRNA^Gly^ prefers G34 rather than A34 that could be potentially modified in I34.

### 2.2. Usage of Codons Gly in Eukaryotes Does Not Show Any Bias

**Figure 2:**
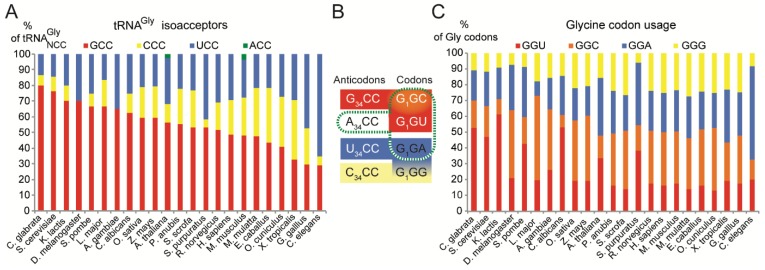
Distribution of tRNA^Gly^ isoacceptors and Gly codon usage in a representative set of eukaryotes. (**A**) Histogram representing the distribution of tRNA^Gly^ isoacceptor genes per organism expressed as percentages (data from http://gtrnadb.ucsc.edu/). (**B**) The codon reading properties of tRNAs^Gly^ are represented with boxes using the colour code used in the histograms of panels A and C. The decoding properties of the rarely found tRNA^Gly^_A34CC_ (modified in I_34_CC) are represented with a dashed green box. (**C**) The histogram represents the codon usage for Gly codons per organism, expressed as percentages (data from http://www.kazusa.or.jp/codon/).

Next we checked whether sparing A34 in tRNA^Gly^ could result from a special codon usage. We examined the ‘Codon Usage Database’ of a list of 23 representative eukaryotic organisms including *Candida glabrata, Candida albicans, Saccharomyces cerevisiae, Kluyveromyces lactis, Drosophila melanogaster, Schizosaccharomyces pombe, Leishmania major, Anopheles gambiae, Oryza savita, Zea mays, Arabidopsis thaliana, Papio anubis, Sus scrofa, Strongylocentrotus purpuratus, Rattus norvegicus, Homo sapiens, Mus musculus, Macaca mulatta, Equus caballus, Oryctolagus cuniculus, Xenopus tropicalis, Gallus gallus, Caenorhabditis elegans* [39]. Despite variations among the organisms, we did not observe any special bias in the usage of Gly codons that would explain the presence of G34 (Figure 2).

As mentioned above, three main tRNA^Gly^ isoacceptors are found in eukaryotes, with GCC, CCC and UCC anticodons. According to the wobble rules, tRNA^Gly^_UCC_ should decode GGA and GGG codons whereas tRNA^Gly^_GCC_ isoacceptor should decode GGC and GGU codons. A third tRNA^Gly^ with CCC anticodon is found to specifically decode GGG codons [29] (Figure 2B). Therefore, the decoding of Gly codons by tRNA^Gly^ is perfectly ensured despite the absence of an A34-containing tRNA^Gly^. It is assumed that such tRNA, if existing, would be deaminated on A34 to form inosine with C, U and A pairing properties (however inefficient with A [31]). Inosine has a greater range of potential base pairs than either adenosine or guanosine. However, I•C and I•U pairs are less stable than G•C and G•U pairs, respectively [40]. In eukaryotes, the G34 anticodon-sparing has been observed for a long time with the exceptional disparity of tRNA^Gly^ [41,42]. In the three kingdoms, for an unexplained reason, G34-containing tRNA^Gly^ read the pyrimidine-ending codons (GGU and GGC) and favor G•U wobble versus I•A, I•C or I•U wobble pairs that would be formed if A34-tRNA^Gly^ modified in inosine would exist. Altogether, these observations suggest that the preference given by tRNA^Gly^ for G34 versus A/I34 cannot be explained by decoding idiosyncrasies of the Gly codons or by economical reasons in terms of tRNA anticodon number. 

### 2.3. Bias of Nucleotide +1 after GGU Glycine Codon

One hypothesis might be that G34 is part of a mechanism of adaptation of tRNA^Gly^ linked to the inherent properties of frameshifting of this tRNA. Many records have described the frameshifting properties of suppressors of tRNA^Gly^_NCC_. They generally frameshift on sequences GGG-N, resulting from expansion of glycine (GGN) codons. 

**Figure 3:**
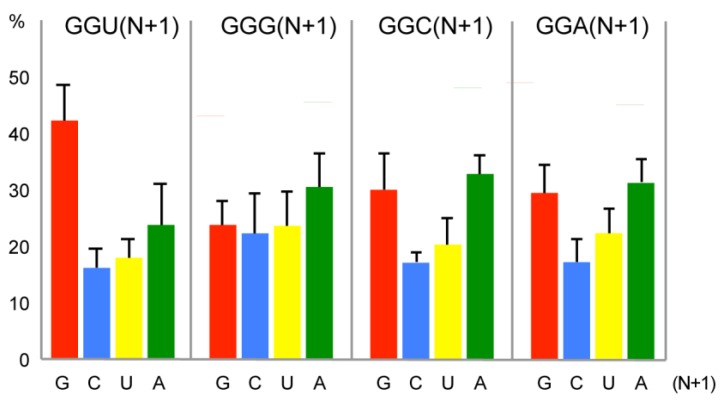
Distribution of nucleotide +1 downstream from the four Gly codons in 23 eukaryotic genomes. 35,246,252 Gly codons were analysed. Results are expressed as percentages. The standard deviation bars are showing the dispersion between the genomes. The *x*-axis corresponds to the nucleotides found at position +1 after the four Gly codons.

**Figure A1:**
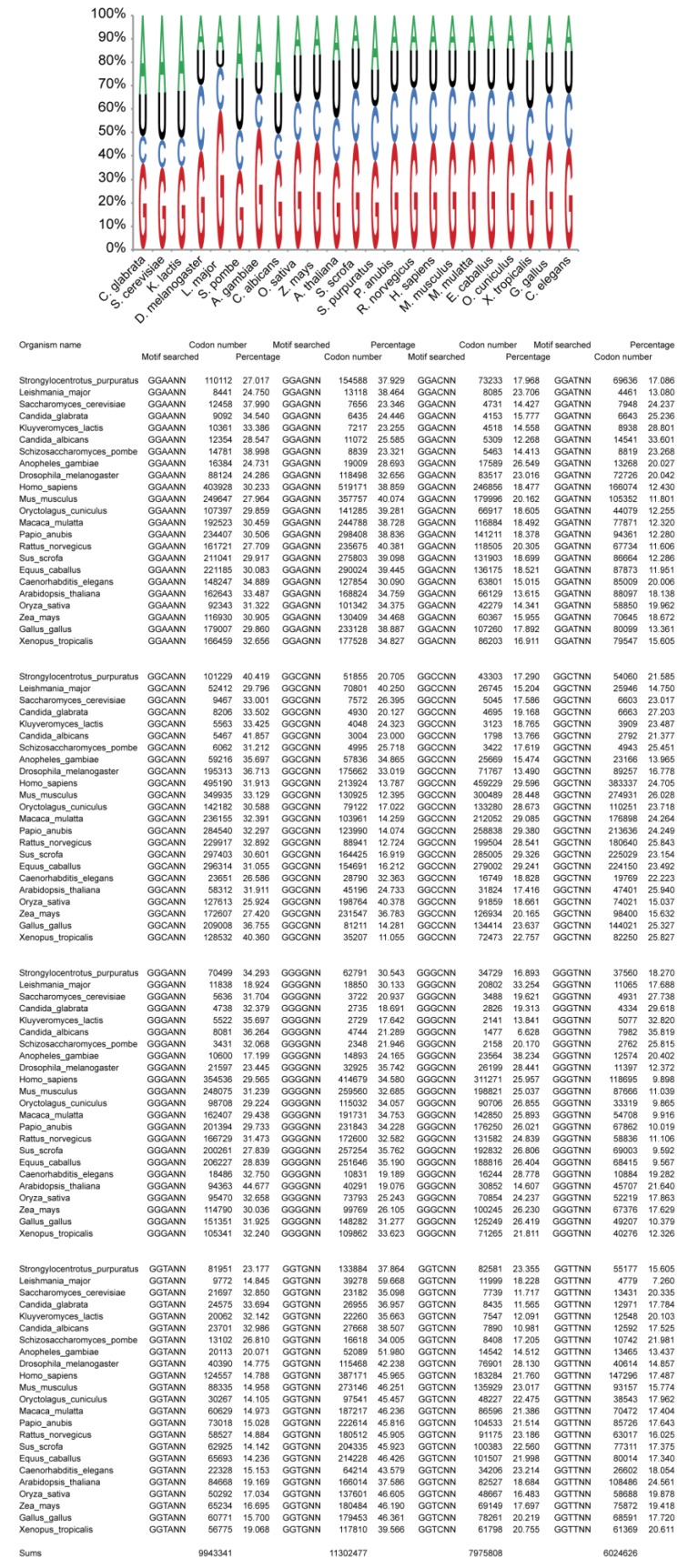
Distribution of nucleotide +1 downstream of GGU Gly codons in each of the 23 eukaryotic genomes. 35,246,252 Gly codons were analysed. Results are expressed as percentages. The *x*-axis corresponds to the organisms while the *y*-axis refers to the occurrence of each nucleotide at position +1. Details of the analysis are shown below the graph.

Therefore, we examined the contexts of Gly codons, focusing on nt+1 that could be involved in +1 frameshifting phenomenon. We analysed all the open reading frames (ORF) of the 23 above-mentioned organisms for their distribution in Gly codons and +1 context. Data processing was performed using a home-written Python script. Coding DNA sequences (CDSs) from genomic sequences of 23 eukaryotic species were downloaded from the NCBI site ftp://ftp.ncbi.nlm.nih.gov/genomes/. 35,246,252 Gly codons were found in the 23 annotated genomes. The histogram of Figure 3 shows the composition of the nt+1 after the four Gly codons, in all the ORFs of the 23 organisms. In the GGU box a clear preference was observed for a G at +1 in 42% of the codons (Figure 3). Normally it is expected to find an equal ratio for the 4 nts with little variation according to the G/C content of the genome. Here, the G preference is specific of the GGU codon and reaches an unusual high value. Although some discrepancies for nt+1 also exist in the other 3 Gly codons, they are less significant (Figure 3). The distribution was examined in each separate organism and the same general pattern of G preference in +1 position of the GGU codons was found with extreme values of 59.7% in *L. major* and 34% in *S. pombe* (Figure A1 in Appendix A).

We also examined the tRNA^Gly^ gene number. The fraction of tRNA^Gly^_GCC_, which decodes GGU codons, varied between 0.29 and 0.80 (Figure 2C) and was well adapted to the Gly codon usage (Figure 2A).

### 2.4. Decoding and Frameshifting Efficiency on Gly Codons

To measure the frame shifting activity on Gly codons we used the second IRES element of CrPV located in the intergenic region (IGR) that initiates using a pseudo knot structure mimicking a tRNA paired with the mRNA. Like the transfer-messenger RNA (tmRNA), the IGR combines features of tRNA and mRNA [43]. However, while tmRNA is involved in the degradation of aberrant mRNAs, the IGR structure is dedicated to translation initiation of the second cistron of the CrPV. A pseudoknot (PKI) of the IGR structurally and functionally mimics the codon-anticodon helix and is recognized by the ribosome in the same way as a cognate mRNA-tRNA codon-anticodon duplex [44]. While tRNAs have low affinity for ribosome in the free form, the IGR binds to the A-site independently of the elongation factor. Then, the pseudo-tRNA is translocated in the P-site, and translation is initiated on the next codon, a GCU in CrPV [6,7,8]. During this process on the ribosome, the IGR undergoes a careful monitoring of its structure, which must perfectly fit with the geometry of a cognate codon/anticodon triplet as found in a tRNA-mRNA complex. For instance, a single mutation that disrupted the pseudo codon-anticodon triplet was shown not to have dramatic consequence on viral protein synthesis. On the other hand, the IGR was perfectly able to start with a stop codon inserted in the pseudo knot [4,5]. Here in this study, the IGR was used as a versatile tool for testing the frameshifting activity induced by the environment of Gly codons. The pseudoknot triplet was reprogrammed with Gly codons and fused with renilla luciferase (R-Luc) ORF in order to initiate in the 0 phase. The insertion of one extra A before the AUG initiation codon of R-Luc induced a +1 frameshift that inactivated R-Luc translation. This construct was used to measure R-Luc activity resulting from the +1 frameshift (+1 frame). By this way we could reprogram the codon and anticodon parts of the IGR in order to explore their reading properties on the ribosome decoding center, and measure the influence of nt+1 on the reading frame maintenance with the R-Luc constructs in 0 and +1 frames.

#### 2.4.1. Decoding and Frameshifting Efficiency of the WT IGR

In a first series of experiment, we tested the activity of the IGR with a wild-type PKI with a G in position +1 of the first codon of R-Luc (G+1 hereafter) and compared it with derivatives with the 3 other nucleotides in +1 (A+1, C+1 and U+1). Transcripts of the constructs in 0 and +1 frame of R-Luc were translated in rabbit reticulocyte lysate (RRL) and the resulting luciferase activities were quantified. 

**Figure 4:**
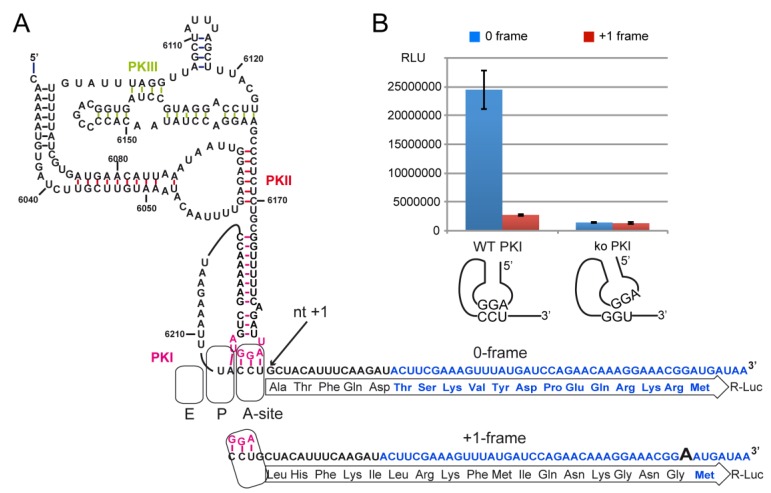
Translation of R-Luc driven by the CrPV IGR and analysis of potential +1-frame translation. (**A**) The secondary structure of the IGR IRES. The IGR is driven translation of Renilla luciferase (R-Luc) fused in the 0-frame or +1-frame. To monitor the +1-frameshifting activity, one extra A nucleotide was added in the R-Luc sequence. Nucleotides and amino acid residues in blue are from R-Luc. The pseudoknot PKI which mimics the codon/anticodon is drawn in pink. The A-, P- and E-site of the ribosome are schematized. Nucleotide numbering is from the CrPV genome. (**B**) Histogram representing the luciferase activities in 0-frame or +1-frame starting from the WT or knockout PKI. Transcripts were synthesized in vitro, denatured/renatured and incubated in Rabbit Reticulocyte Lysates at 30 °C for 2 h. Translation of R-Luc was measured by measuring the bioluminescence produced with a Dual-Glo luciferase assay (Promega) in a luminometer. Translation efficiencies are represented as raw bioluminescence activity (Relative Light Units or RLU) and resulted from at least 3 independent experiments. Standard deviations are shown.

We first measured the R-Luc activity of the wild-type (WT) PKI with the complementary sequence AGG/CCU mimicking a tRNA^Pro^_AGG_ annealed to a CCU Pro codon. In parallel, a negative IGR control was constructed with a disrupted PKI exhibiting a GGU codon instead of CCU [5]. The disruption strongly diminished the 0 and +1 frame translation activity to a level of 4% of the activity of the WT IGR in 0-frame (Figure 4). The background was identical in 0 and +1 frame, and could account from the spontaneous cap-independent translation activity typically measured with RRL extracts. This background was subtracted from the measured activities in all the following experiments.

**Figure A2:**
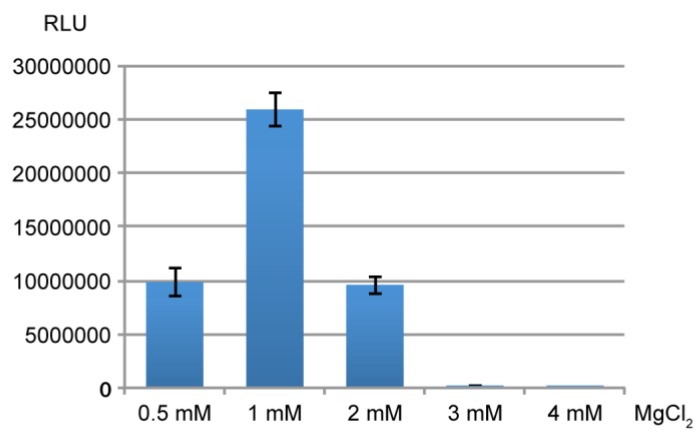
The effect on translation of the Mg^2+^ concentration added to the extracts. R-Luc activity was measured in the 0 frame. Translation efficiencies are represented as raw bioluminescence activity (Relative Light Units or RLU) and resulted from at least 3 independent experiments. Standard deviations are shown.

With the WT IGR, a spontaneous frameshifting activity was observed when comparing the activity of R-Luc in 0 and +1 frame. This is consistent with other studies performed on the IGR of other dicistroviruses that showed translation of an overlapping ORFx in the +1 frame [44]. The R-Luc activity in +1 frame reached 7% of that of 0 frame, quite similar to the activity previously reported [44,45,46]. We also observed that the ratio of synthesis of the two frames is sensitive to the Mg^2+^ concentration and translation conditions as described [47]. Optimal Mg^2+^ concentration to add was found to be 1 mM in our activated RRL translation extracts. With 0.5 mM or 2 mM Mg^2+^ the extracts were less active and less specific for frame selection. They were entirely inactive above 2 mM (Figure A2).

**Figure 5:**
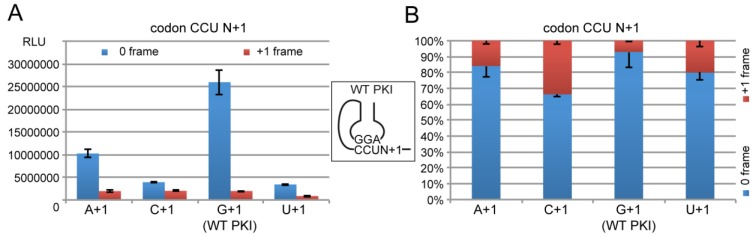
The effect on translation of the first nucleotide downstream the wild-type pseudo knot (PKI). (**A**) Histogram representing the luciferase activities of the synthesized R-Luc in 0-frame or +1-frame. Translation efficiencies are represented as raw bioluminescence activity (Relative Light Units or RLU) and resulted from at least 3 independent experiments. Standard deviations are shown. The construct with a G+1 corresponds to the WT PKI. (**B**) The same data represented as a 100% stack bar graph.

Then we analysed the impact on translation efficiency of the substitutions of the first nt after the WT CCU Pro codon of PKI. These mutations change the first amino acid and may potentially affect translation initiation efficiency and frame selection. Dicistroviruses are able to initiate with different amino acids. Plautia stali intestine virus (PSIV) starts with Gln (CAA), Israeli acute paralysis virus (IAPV) with Gly (GGC) and many other dicistroviruses start with Ala (GCU/C/A) [48]. Here we generated the 3 constructs of the WT PKI with an A, C or U in +1. The resulting new codons were ACU, CCU and UCU leading to Thr, Pro and Ser changes, respectively. These 3 amino acids are considered to be non-destabilizing residues according to the N-end rule and therefore should not affect differently the half-life of the translated products [49]. However, when comparing the R-Luc activities, we could observe significant variations with the 4 constructs. The most active construct in 0 frame was the WT IGR with G in +1 downstream of non-coding Pro codon of PKI. This result suggests that evolution has already selected the most efficient amino acid to initiate de synthesis of the viral protein. Compared to the WT PKI with a G+1, the other constructs with A+1, C+1 and U+1 exhibited 39%, 15% and 13% of activity, respectively (Figure 5). Therefore the nt in +1 significantly impacts translation initiation despite its location outside of PKI. In the cryo-EM structure of the CrPV IGR in complex with *K. lactis* 80S, an additional base pair is observed between G+1 and the ribosomal base C1273 that is usually interacting with the third base pair of the anticodon during standard decoding [9]. Remarkably, all IGR-IRES sequences obtained to date encode a guanine residue at this position with the only exception being the PSIV-IRES [48]. This conservation correlates with the function of G+1 in mRNA frame selection [47], and the variations of translation efficiency here observed with the 3 variants.

Interestingly, the consequent decreases of activity in 0 frame were not correlated with increases of activity in the +1 frame that would suggest competition for ribosome site selection in the 0 or +1 frame (Figure 5). However, the ratio of R-Luc activity in +1 and 0 frame showed significant variations from 0.07 for the WT IGR to 0.19, 0.51 and 0.25 for the variants with A+1, C+1 and U+1, respectively. These data confirmed that nt+1 is involved in frame selection [47] although the loss of activity in 0 frame was not balanced by an increase of activity in the +1 frame. The molecular mechanism of this frameshifting is not yet clearly understood, but it has been proposed that on the IGR, the ribosome is oscillating between 0 and +1 frames and the frame is locked when the cognate tRNA binds [14]. At this step, the guanine residue in +1 might play a critical role in stabilizing the conformation more suitable for the 0 frame. Therefore, the tRNA arrival rate would dictate the choice of the 0 and +1 frame to translate [14]. This is consistent with the fact that tRNA^Ala^_AGC_ for the 0 frame is an abundant isoacceptor whereas tRNA^Leu^_UAG_ for the CUA codon of +1 frame is a relatively rare isoacceptor [29].

#### 2.4.2. Decoding Efficiency of the IGR Programmed with GGU Gly Codon

**Figure 6:**
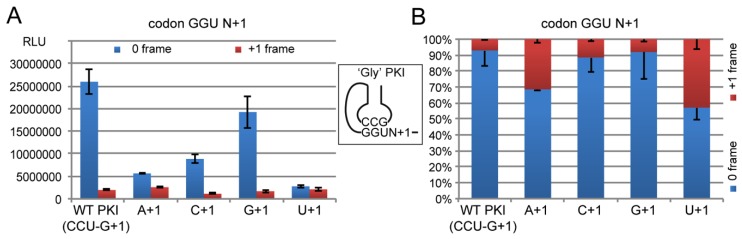
The impact on translation of the first nucleotide downstream of the PKI reprogrammed with a Gly GGU codon. (**A**) Histogram representing the luciferase activities of the synthesized R-Luc in 0-frame or +1-frame. Translation efficiencies are represented as raw bioluminescence activity (Relative Light Units or RLU) and resulted from at least 3 independent experiments. Standard deviations are shown. The first construct corresponds to the WT PKI. (**B**) The same data represented as a 100% stack bar graph.

First, we tested PKI constructs programmed with a GGU Gly codon. As mentioned earlier, GGU codon is decoded by tRNA^Gly^_GCC_. We replaced the CCU codon of the WT PKI by the GGU Gly codon and to preserve the complementarity with the pseudo-anticodon of PKI, we inserted the GCC sequence in the opposite strand (Figure 6). The resulting codon-anticodon minihelix contained an equivalent ‘G34•U3′ wobble pairing, a combination never tested in the IGR. Indeed, previous mutagenesis studies established the essential role of the correct base pairing of the tRNA/mRNA mimicry in PKI of the IGR [4,5]. As the IGR mimics a tRNA paired with the mRNA, it is possible to test all the codon/anticodon combinations of interest, including non-canonical base pairing.

As before, the reprogrammed PKI was tested with the 4 nts in position +1 and luciferase activity was assessed in 0 and +1 frame (Figure 6). Of the 4 constructs, GCC/GGU-G was the most efficient with 74% of the R-Luc activity measured with the wild-type AGG/CCU-G construct. Therefore a construct with ‘G34•U3′ wobble pair is well recognized by the ribosome in the presence of a G in +1. The 3 other constructs exhibited severe decreases of the activity in 0 frame with 21, 34 and 11% of WT activity for A, C and U in +1, respectively (Figure 6). These results confirmed the critical role of nt+1 for the IGR activity even with a PK reprogrammed with a Gly codon.

#### 2.4.3. Decoding Efficiency of the IGR Programmed with Other Gly Codons

**Figure 7:**
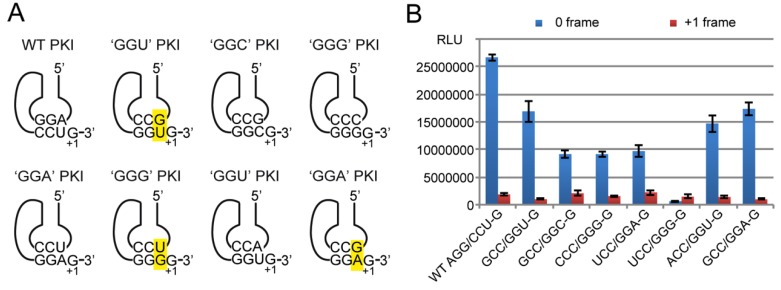
The effect on translation of reprogramming the PKI with different Gly codon-anticodon combinations. (**A**) Schematic representations of the different RNAs tested. Non Watson-Crick base pairs are highlighted in yellow. (**B**) Histogram representing the luciferase activities of the synthesized R-Luc in 0-frame or +1-frame. Translation efficiencies are represented as raw bioluminescence activity (Relative Light Units or RLU) and resulted from at least 3 independent experiments. Standard deviations are shown. Construct names are described as XXX/YYY-G where X represent anticodon triplets, Y codon triplets, all with a G in +1 and written in the 5′-3′ direction. The first construct corresponds to the WT PKI.

In addition to the IGR programmed with the GGU codon, we constructed variants with the other Gly codons. A first variant contained a GGU Gly codon (with G+1) annealed to an ACC anticodon instead of GCC. The (ACC/GGU-G) variant mimics recognition by the absent tRNA^Gly^_A34CC_, which in eukaryotes is specifically replaced by the anticodon with a G34. Here a regular Watson-Crick pairing ‘A34-U3′ is expected to form in the ACC/GGU-G variant instead of the ‘G34•U3′ wobble pairing found in the GCC/GGU-G construct. The RNA with the Watson-Crick ‘A34-U3′ pair was nearly as active as the construct with ‘G34•U3′ wobble pair (55% and 65% of the activity of the WT IGR, respectively) (Figure 7). Thus, the absence of tRNA^Gly^_A34CC_ is not due to a fundamental structural or functional defect hampering its function in translation. Nevertheless, the RNA with ‘A34-U3′ pair is 10% less active than the ‘G34•U3′ whereas the frameshifting activity is increased to 9% (versus 7% for the ‘G34•U3′ construct). Therefore, the GCC anticodon forming a wobble ‘G34•U3′ with the GGU codon is the most efficient and faithful combination.

Four other constructs were generated to mimic the other codon-anticodon combinations formed during codon Gly decoding. Transcripts GCC/GGC, CCC/GGG, UCC/GGA, and UCC/GGG (all with G in +1) were constructed in order to test initiation on GGC, GGG and GGA codons in the PK, respectively. The 3 first transcripts exhibited 35% of residual activity in 0-frame translation whereas the fourth one, the UCC/GGG variant, had lost nearly all its activity in frame 0, which was below the level of the +1 frame activity. The low activity of the UCC/GGG variant with a ‘U34•G3′ wobble pair may be surprising since the GCC/GGU variant with the reverse ‘G34•U3′ pair exhibited 65% of the WT activity. This result confirms that G•U and U•G pairs are not isosteric [50] and confer a non-functional geometry to the IGR in the ‘U34•G3′ orientation. Usually U34-containg tRNAs utilize diverse modifications to stabilize tautomer conformations suitable for the ribosomal A-site [51]. However here, the transcripts of the IGR are devoid of any modification and therefore the PKI ‘anticodon loop’ cannot achieve the suitable conformation change. This might explain the drastic effect on the activity of the 0-frame while the frame-shifting activity was kept nearly constant.

Overall, the data collected with the 5 constructs are showing that the presence of two strong G=C pairs in codon positions 1 and 2 do not guarantee the full activity to the IGR. The more active constructs are exhibiting a weak third pair such as a wobble pair in the ‘G34•U3′ orientation or an A-U pair. Three G=C pairs are reducing the activity as well as the reverse wobble pair ‘U34•G3′.

To confirm this observation, an additional construct GCC-GGA with a non-Watson-Crick pair at position ‘G34•A3′ was constructed and tested. The construct was as active as the best Gly construct (Figure 7). G•A pairs are supposed to bring flexibility to RNA structures [52,53,54,55]. Up to six dominant modes of G•A pairing have been reported in RNA molecules comprising ribosomes, riboswitches, ribozymes, tRNAs, mRNAs, etc. [56] and the geometry of a G•A pair was solved at the central position of the codon-anticodon helix in the 70S ribosome [57].

Altogether, the data show that in the Gly codon context, the presence of more than two G=C Watson-Crick pairs in the PK is deleterious for the translation initiation activity. The presence of A-U at the third codon position is tolerated, but a weaker G•U wobble pair or a G•A non-Watson Crick pair is conferring the highest and faithfulness translational activity to the IGR. All these results are consistent with our knowledge about the ribosomal A-site. It recognizes precisely the geometry of codon-anticodon base pair at the first two positions but monitor the third, or wobble position, less stringently, allowing diverse unusual base pairs to interact [58,59].

According to the energies of the codon-anticodon minihelices calculated following the Turner values [60], the Gly amino acid decoded by GGN codons belongs to the ‘STRONG’ group with high energy (average −4.6 kcal/mole) in their codon-anticodon duplexes [28]. Such high energy represents a risk of miscoding by binding to other GC-rich non-cognate aa-tRNAs. Therefore, the translation apparatus evolved to minimize the differential thermodynamic contributions of the codon-anticodon interactions. It results that the structures of tRNAs harboring too strong base-pairing binding capacity have been ‘tuned down’ during evolution for optimal and uniform translation. Consequently, the binding of natural tRNAs on the ribosome is very smilar whatever the GC/AU composition of the codon-anticodon duplex [27]. One critical feature that modulates the binding energy of codon-anticodon duplexes is the interaction between bases 32 and 38 in the tRNA anticodon loop. Classical Watson-Crick base pair constraints the anticodon loop structure and antagonize the canonical conformation of the codon-anticodon triplet, thereby weakening the corresponding binding energy. Watson-Crick base pairs in 32–38 are often associated with GC-rich anticodons, whereas weaker non-canonical pairs are found in tRNAs with AU-rich anticodons where they allow optimal anticodon conformation for pairing with the codon. By this way, the differential thermodynamic contributions of all the codon-anticodon interactions is averaged, resulting in a rather uniform binding of tRNAs to the ribosome [28,61,62]. Here in our experiments the high binding energy of Gly codons inserted in the PK couldn’t be balanced by a compensatory change in the IGR since it remained unchanged. This may explain that 2 G=C bp already reached an energy limit above which any additional Watson-Crick bp would decrease translation initiation efficiency. Above this limit, the PK probably possess too much energy compared to the external stabilizing grip energy for the ribosome required during translation, resulting in a possible rejection from the ribosomal decoding site.

#### 2.4.4. Frameshifting Activity of the IGR Programmed with GGU Codons

During translation, tRNAs move from the A site on the ribosome to the P site and then from the P site to the E site together with the mRNA. While moving, the codon-anticodon interactions remain intact through the A, P and E sites. A critical checkpoint for the maintenance of ribosome fidelity is located in the A-site where the geometry of codon–anticodon helix is tightly monitored by the three universally conserved and essential nts A1492, A1493, and G530 (bacterial numbering) of 16S rRNA [63,64]. This is achieved by intimate interactions with the minor groove of the first two bp of the codon-anticodon helix, thus sensing Watson-Crick base-pairing geometry and discriminating against near-cognate tRNA. The third (wobble) position of the codon is free to accommodate certain non-canonical base pairs. At this level, C1054 of 16S rRNA stacks against base 36 of the tRNA, which also interacts with G530, and ribosomal protein S12.

Additionally, natural modifications located in the anticodon loop (nt 37) and anticodon itself (nt 34), stabilize tRNA-codon interactions and increase decoding fidelity, or expand decoding capacity (nt 34). Downstream of the A-codon, nucleotides +7, +8 and +9 (the A of the AUG being nt+1) of the mRNA are held in place by a combination of hydrogen bonding and continuous aromatic base stacking involving, among others, C1054 from helix 34 of 16S rRNA, further stabilizing the network [65]. Altogether, data suggest that during elongation, the mRNA is stabilized and aligned at the entrance of the decoding center by a network of interactions with the ribosome [66,67].

Recently, a significant variation of this stabilizing network was observed in the cryo-EM structure of the IGR element of CrPV in complex with *K. lactis* ribosome [9]. Instead of the usual interaction between the C1054 (C1273 in *K. lactis*) and codon base 3, a unique pairing is observed with nt+1 of the first translated codon of the viral protein. This exceptional base pair results from the rotation of the base around its glycosidic bond causing the redirection of C1273 towards the first base of the first coding codon of the CrPV RNA [9]. Remarkably, with the exception of the IGR of PSIV [48], all the IGR sequences obtained to date exhibit a guanine residue at this position suggesting that the interaction C1273-G+1 may be mostly conserved in the dicistrovirus family. A sub-class of IGR elements synthesize in addition to the main ORF2 an overlapping hidden polypeptide (ORFx) in the +1 reading frame. Therefore it was proposed that the extra base pair C1273-G+1 might be an important regulatory element downstream of the ribosomal A site that could be involved in the frame selection by the IGR element [44,45,47]. In Israeli Acute Paralysis Virus (IAPV) IGR, the ORFx synthesis is initiated immediately downstream of the IGR, using a mechanism of frame selection probably dictated by the kinetics of tRNA binding in the first 0 or +1 frame codon as measured for the CrPV IGR [14].

Here in response to G+1 substitutions in the wild-type CrPV IGR, a decrease of translation of the 0 frame was observed (Figure 5 and Figure 6). Translation of the +1 frame was also decreased but to a lesser extent, resulting in a global loss of fidelity in the frame maintenance as shown by the vertical stacked bar graphs (Figure 5B and Figure 6B). Three constructs (AGG/CCU-C, GCC/GGU-A and GCC-GGU-U) exhibited frameshifting activity higher than 30% of the total R-Luc activity (Figure 4B and Figure 5B).

Therefore, the C1273-G+1 interaction contributes to efficiency and fidelity of the translation process, assisting the PKI element in the presentation of the adjacent first codon in a proper conformation to the first incoming tRNA. The IGR IRES has a dynamic structure that is able to adopt upon ribosome-binding all the conformations of the aminoacyl-tRNA from the initial binding to the fully accommodated stage after translocation into the P-site [12]. Our data and previous reports [9,12] clearly show that nt+1 plays a critical role when interacting with C1273 (C1054 in *E. coli* ribosomal RNA) in a conformation reminiscent of a hybrid tRNA state [44,45,47]. Although this interaction has never been observed in the large number of described ribosomal complexes with tRNAs or ASLs (for a full set of references [9]), one cannot exclude that C1273 (C1054) interacts with G+1 during standard decoding. C1273 may flip from the interaction with G+1 during the initial binding to stacking on the wobble base 34 as previously reported [50,51]. Another evidence of the importance of C1273 (C1054) during ribosome decoding is coming from early genetic studies. Mutants of C1054 with reduced translational accuracy were isolated, causing non-specific read-through of stop codons as well as enhanced +1 and −1 frameshifting [63].

Based on our systematic mutational analyses, the most likely scenario is that ribosomes recruited to the IGR initiate in the 0-frame or shift in the +1 frame depending on the codon-anticodon and downstream +1 nt. The presence of C1273 (C1054) would stabilize the conformation of PKI by interacting with a G in +1. Here evidence is presented which shows that the IGR activity is favored when it is programmed with a U-ending codon (AGG/CCU, GCC/GGU, ACC/GGU) and a G in +1. It includes the Gly GGU codon with G in +1. Our first finding that a G preferentially follows a GGU codon (Figure 3) can now be explained in terms of decoding efficiency and reading frame fidelity in the context of the PKI element of the IGR element. Indeed, although the IGR mimics the tRNA shape, it lacks some critical structural elements such as the base pair interaction between nts 32 and 38 which usually fine-tunes the loop structure and harmonize tRNA affinity for ribosomes [68,69,70,71] and the proofreading step on the ribosome by eEF1A. Additional experiments are now required to validate the conclusions using Gly codons located outside of the PKI in the coding sequence of R-Luc. This will allow testing natural modified tRNAs in the context of competition of a total tRNA extract, including the phenomenon of miscoding by near-cognate tRNAs when that was not the case with the IGR reprogrammed element.

## 3. Materials and Methods

### 3.1. Data Processing

Data processing was performed using a Python script. Coding DNA sequences (CDSs) from genomic sequences of 23 eukaryotic species were downloaded from the NCBI site ftp://ftp.ncbi.nlm.nih.gov/genomes/.

The first part of the script checked all CDSs of each species. It removed CDSs from the non-nuclear genome, annotated as pseudogenes and having a nucleotide length not a multiple of three. CDSs were translated taking into account the genetic code of each species and the translation exceptions like the selenocysteine UGA codon. CDSs were excluded if their protein sequence (i) did not begin with an AUG or an alternative initiator codon, (ii) did not end with a stop codon and (iii) contained a stop codon within their coding phase.From the DNA sequences of the retained CDS set, the second part of the script used a sliding window (window size of 6 bases and step size of 3 bases) to search four nucleotide patterns. These patterns correspond to the four glycine codons, GGU, GGG, GGC and GGA, followed by all base possibilities in position +1 and unknown bases in positions +2 and +3. For example, patterns GGTANN, GGTGNN, GGTCNN and GGTTNN were searched. The occurrence of the four given patterns was counted to calculate the relative percentage of each pattern. The output of the script was a csv file.

### 3.2. Construction of the IGR-luciferase Reporters

**Figure A3:**
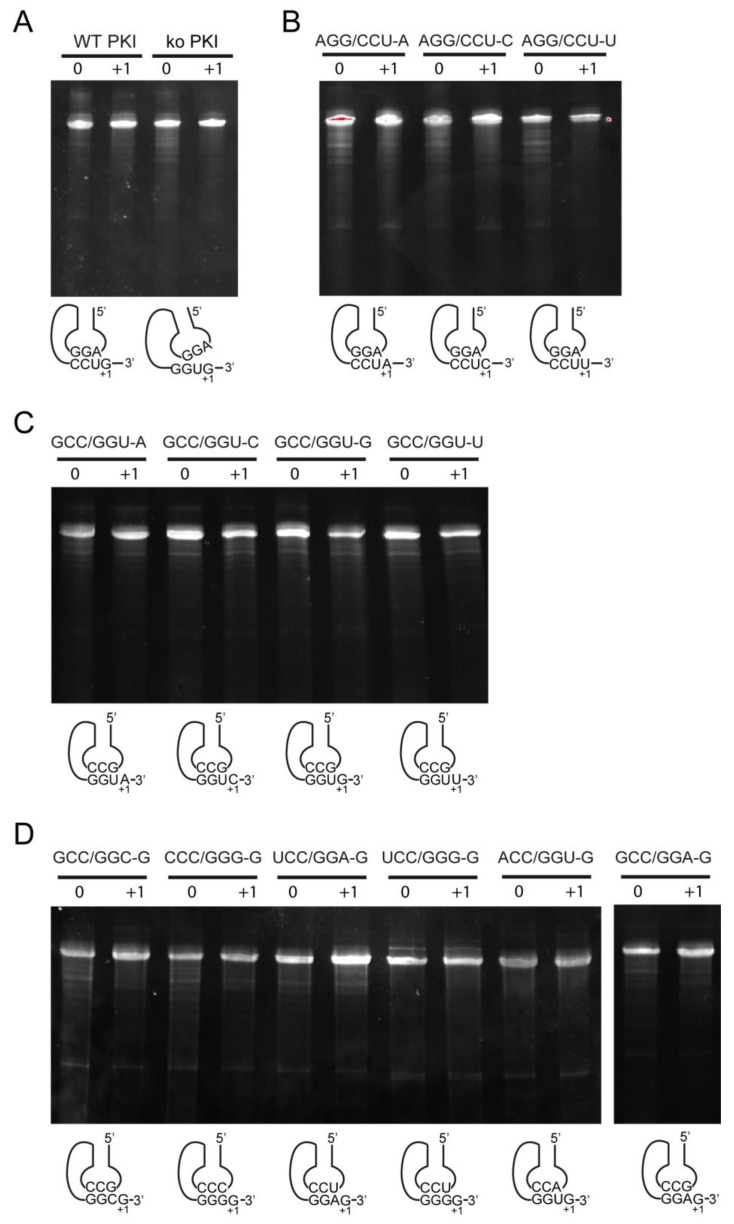
Control of the integrity of the RNA constructs used in the study. RNA samples (0.4–0.6 μg) were fractionated by 4% denaturing PAGE and stained with ethidium bromide. (**A**) RNAs used in Figure 4; (**B**) RNAs used in Figure 5; (**C**) RNAs used in Figure 6; (**D**) RNAs used in Figure 7.

The PK mutants were constructed by PCR amplification of the CrPV IGR coding template using a sense primer (5′GGATATTAATACGACTCACTATAGG*CAAAAATGTGATCTTGCTT*3′) appending the T7 RNA polymerase promoter (underlined sequence) to the IGR structure (italic) and an antisense primer (5′**CGAAGTATCTTGAAATGT***AGCAGGTAAATTTCTTAGGTTTTT CGACTACCTAATCTGAAA*3′) where nts from the IGR are italicized, those forming the pseudo codon/anticodon are underlined and bold nts are complementary to Renilla Luciferase gene (Integrated DNA Technologies, Coralville, IA, USA). To generate the IGR variants, a PCR mixture containing 0.02 ng/µL of CrPV IGR-containing template plasmid, 0.2 µM of sense and antisense primer (Integrated DNA Technologies), 0.2 mM of each dNTP (ThermoFisher, Waltham, MA, USA), 0.04 U/µL of Phusion DNA polymerase (ThermoFisher) and the corresponding buffer at the recommended concentration were subjected to an initial denaturation step of 2 min at 95 °C followed by 25 cycles of: 30 s at 95 °C, 30 s at 55 °C, 1 min at 72 °C, and terminated by a final extension of 10 min at 72 °C. PCR products were fractionated on a 1% agarose Tris-acetate gel and the PCR product was excised from the gel and eluted by PCR Clean-Up System” kit (Promega, Madison, WI, USA) before quantification with a Nanodrop (Thermo Fisher Scientific, Waltham, MA, USA). This PCR product was then used as primer in a second PCR reaction using as template the Renilla Luciferase gene (pRLuc). The second PCR was performed in the same conditions as above but using 0.02 ng/µL of pRLuc, 0.07 µM of the first PCR product (the region of the antisense primer complementary to R-Luc is in bold) and 0.2 µM of RevRLuc primer (5′GAATTATTGTTCATTTTTGAGAAC3′). To measure the luciferase activity resulting from a frameshift of +1 nucleotide, the second PCR amplification was performed on template pRLuc+1, which exhibits one extra A before residue Met 14 of Renilla Luciferase. Finally, the second PCR products were purified on a 1% agarose gel, and quantified as above. IGR-RLuc reporter mRNAs were synthesized from the second PCR product by in vitro transcription using recombinant T7 RNA polymerase as described previously [72,73]. After transcription, unincorporated nucleotides were trapped on a G-25 column. RNA transcripts were then phenol-extracted and ethanol precipitated. RNA pellets were resuspended in water and their concentration was determined by absorbance measurements. Figure A3 shows the RNA integrity of the constructs used in the study.

### 3.3. In vitro Translation in Rabbit Reticulocyte Lysates

**Figure A4:**
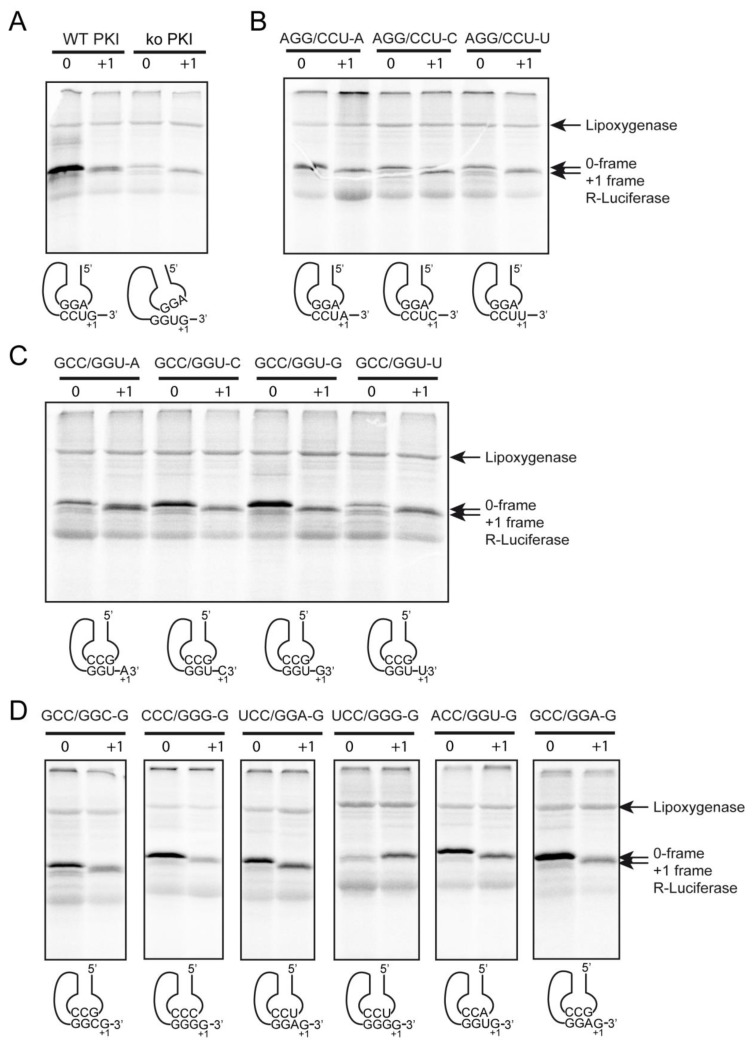
Analysis of the IGR-dependent translation products in vitro. For each construct, the 0- and +1-frame products were analysed. The reporter constructs were incubated in RRL translation extracts at 30 °C for 60 min in the presence of [^35^S]Met. Translation experiments were performed in untreated RRL containing endogenous lipoxygenase mRNA as well as globin mRNA in large amount (the globin band is not shown). Translation products were visualized by autoradiography after resolving on a 10% SDS PAGE.

Translation reactions were performed in RRL extracts as previously described [74]. Prior translation, the IGR RNA was heated at 95 °C for 3 min, then cooled to room temperature during 5 min and placed on ice. Translation reactions were incubated at 30 °C for 60 min and included 100 nM of each IGR-reporter mRNA transcript and 10.8 μCi [^35^S]Met. Aliquots of translation reactions were analyzed by 15% SDS-PAGE (data not shown) and luciferase activities were measured with the Promega dual-luciferase assay, using a Promega luminometer. Figure A4 shows the radioactive translation products after separation by SDS PAGE and autoradiography.

## 4. Conclusions

Here we combined experimental and bioinformatics approaches to analyze the Glycine codon usage with the 3′ downstream nucleotide (nt+1). A clear preference for a G in +1 was detected after the GGU codon. When a G residue was in +1 of an IGR construct reprogrammed with a GGU codon, the translational activity of the reporter R-Luc was improved in the 0 frame and frameshifting activity in the +1 frame dropped to a minimum. As GGU codons are read by tRNA^Gly^_GCC_, our data suggest that G34 together with G+1 is there to prevent +1 frameshifting. Altogether, the results show that translation efficiency of Gly GGU codon by tRNA^Gly^_GCC_ is modulated by the first nucleotide of the adjacent codon, acting simultaneously on the frameshifting activity.

Glycine is a member of a 4-codon box family whose two first nts are forming strong G=C pairings. Commonly, the Gly box is read by up to three different isoacceptors. What is remarkable in this box is that tRNA^Gly^_A34CC_ has been systematically banished from nearly all organisms, being replaced by tRNA^Gly^_G34CC_ with decoding properties for GGC and GGU codons. The other boxes usually exhibit a tRNA_ANN_ that is subsequently deaminated in inosine with extended decoding properties for C, U and A-ending codons. The reason of the exceptional A34 sparing in the Gly box family is not clearly understood but several hypotheses have been proposed. For instance, it was shown that tRNA^Gly^_A34CC_ was an extremely poor substrate for the deaminase enzyme (adenosine deaminase acting on tRNA; ADAT) responsible for I34 formation [75]. This might be due to an unusual structure of the loop. Molecular dynamics (MD) simulations revealed that an A34 could be an important source of structural instability in the anticodon stem-loop of tRNA^Gly^ [75]. Moreover, solution nuclear magnetic resonance (NMR) analyses on different tRNA^Gly^ anticodon arms showed that they do not form the classical U-turn motif seen in tRNA anticodon loops [76]. Together, these results indicate that the singular tRNA^Gly^ anticodon loop structure might be a potential cause for the selection of G34 versus I34 in tRNA^Gly^. An unmodified A34 would only be able to decode U-ending codons while tRNA^Gly^_G34CC_ is able to decode G and U-ending codons. Beyond this idiosyncrasy, it is worth pointing out that evolution has assigned to tRNA^Gly^_G34CC_ and whole tRNA^Gly^ family multiple extra-translational roles, including transcriptional regulation with the T-box regulatory elements [77] and cell wall biosynthesis [78].

## Figures and Tables

**Table 1:** Identity of nucleotide 34 in tRNAs in the 3, 4 and 6 codon boxes *.

tRNAs Specific for	Total Number in Eukarya ^1^	With G34	With A34	With C34	With U34
Ala, Arg, Ile, Leu, Pro, Ser, Thr and Val	80,060	**3063**	**40,726**	14,194	22,077
Gly	13,347	**6636**	**767**	2328	3616

^1^ from http://gtrnadb.ucsc.edu/ [29]; * Excluding in the 6-codon boxes the tRNAs for the 2-codon boxes.

## Abbreviations

ASL	Anticodon Stem Loop
CDS	Coding DNA sequence
CrPV	Cricket Paralysis Virus
IGR	InterGenic Region
IRES	Internal Ribosomal Entry Site
nt	nucleotide
ORF	Open Reading Frame
PKI	Pseudoknot I
R-Luc	Renilla Luciferase
RRL	Rabbit Reticulocyte Lysate
